# EnsembleAge clock: a reliable and robust epigenetic age clock service reveals epigenetic age acceleration in opioid-overdosed brains

**DOI:** 10.1186/s12864-025-12271-9

**Published:** 2025-12-06

**Authors:** Akshay Anand, Yash Agarwal, Tanisha Gupta, Jason Lin, Mirna Ghemrawi, Glenn S. Gerhard, Hayan Lee

**Affiliations:** 1 Cancer Epigenetics Institute, Nuclear Dynamics and Cancer Program, Fox Chase Cancer Center, Philadelphia, PA USA; 2 College of Computing, Data Science, and Society, University of California, Berkeley, CA USA; 3 Department of Computer Science, University of California, Los Angeles, CA USA; 4 College of Computing, Georgia Institute of Technology, Atlanta, GA USA; 5 Computational Biology, Brown University, Providence, RI USA; 6 Center for Forensic Science Research and Education, Florida International University, Horsham, PA USA; 7 Department of Medical Genetics and Molecular Biochemistry, Lewis Katz School of Medicine, Temple University, Philadelphia, PA USA

**Keywords:** Epigenetic age clock, DNA methylation, Machine learning, Aging biomarker

## Abstract

Age is a major risk factor for various diseases, such as cancer, cardiovascular conditions, and neurodegenerative diseases. However, chronological age, the simple number of years one has lived, does not capture individual health differences, prompting the development of methods to accurately estimate biological age instead of relying on chronological age. One of the major molecular approaches exploits DNA methylation (DNAm), which is an essential epigenetic modifier for regulating gene expression, cell differentiation, and aging. DNAm-based aging clocks have been developed to predict biological age, but the prediction is highly dependent on training data, including organs and assay technologies. To address these clocks’ high variance, we present two EnsembleAge clocks, leveraging eight previously developed DNAm clocks, harnessing the strengths of each methylome age clock, smoothing out individual variances, and providing a more robust estimation of biological age. We trained our EnsembleAge clock models using DNA methylation data from nine organs in the Genotype-Tissue Expression (GTEx) dataset. Our EnsembleNaive clock model achieved the lowest median absolute error (MeAE) of 4.04 years in whole blood. The EnsembleLR model demonstrated the lowest MeAE of 6.35 years across multiple tissues, including breast, lung, muscle, ovary, prostate, testis, and colon. The significant reduction in MeAE underscores its high practical value in clinical and forensic applications, especially in contexts where epigenetic changes are subtle. We further applied our models to four public datasets representing diverse biological applications, including administered short-term medical opioid use (GSE151485) and long-term opioid overdose (GSE164822). Our model reveals over 10 years of age acceleration in opioid-overdosed brains but no significant epigenetic age acceleration when opioid usage was well administered. Our EnsembleAge clock models are also implemented as a web service, allowing users to conveniently upload their DNA methylation data and receive predictions of their biological age. This empowers individuals to track their biological/epigenetic age over time, mitigating the effect of variance and promoting healthy aging and a beneficial lifestyle. Our EnsembleAge clock service is available at https://ensemble.epiclock.app/.

**Supplementary Information:**

The online version contains supplementary material available at 10.1186/s12864-025-12271-9.

## Background

Biological age captures interindividual variations and susceptibilities to age-related diseases and mortality better than chronological age does [1]. Unlike chronological age, which simply measures the number of years a person has lived, biological age reflects the combined impact of factors such as diet, physical activity, genetics, and overall health [2]. Biological age is influenced by underlying aging and biological processes, making it a better determinant of disease risk, progression, and mortality [3]. While the genetic information encoded in DNA is identical across all cell types, the epigenetic information encoded around DNA varies by cell type, regulating biological processes [4]. Epigenetic regulators are expected to change over time, and dysfunction of these cellular processes can lead to disease. DNA methylation (DNAm), the covalent transfer of a methyl group to the C-5 position of cytosine in a CpG dinucleotide, is one of the major sources of epigenetic information [5]. Many DNAm-based clock models rely on datasets generated via the Illumina Infinium methylation array, a widely used platform in epigenetic research. This array examines DNA methylation at half a million (HM450K) to one million (EPIC) specific CpG sites across the genome, providing detailed insights into epigenetic changes associated with aging and disease [6].

The initial DNAm aging clock, developed by Horvath in 2013, produced biological age estimates that correlated with chronological age above r = 0.90 across multiple tissues and a wide age range [7]. The Horvath clock model introduced DNAm age acceleration, a metric for indicating whether biological age prediction exceeds chronological age, suggesting that one can be biologically older than their chronological age [1]. Despite its potential utility and performance, conceivable biases—particularly related to the distribution of sample ages or the overrepresentation of specific tissue types in the training datasets—may arise due to inherent limitations in the available training data.

It is, therefore, essential to maintain an unbiased age distribution of samples and tissue/organ types in training datasets. Organ- and tissue-specific DNA clocks should increase precision and accuracy. For example, brain-specific epigenetic clocks have been developed to serve brain aging, Parkinson's, and Alzheimer’s disease populations [8]. The Hannum clock, designed specifically for whole blood, shows a strong correlation with chronological age, similar to Horvath’s clock (r > 0.90) [9]. Horvath’s Skin and Blood clock, tailored for skin and blood tissues and cells, also performed well in brain, liver, and bone, and was shown to accurately track the association between cell aging and proliferation, making it a highly sensitive and robust age estimator [10]. Zhang’s 2019 pan-tissue aging clock also showed high accuracy in endometrium and saliva samples [11].

Instead of solely estimating epigenetic age, some clocks incorporate clinical phenotypes—such as age, gender, BMI, and smoking history—to improve mortality prediction [12]. The PhenoAge clock focused on healthspan, all-cause mortality, and specific diseases. It was trained on blood DNAm data with 513 CpG features. This clock exhibited significant age acceleration for individuals who smoked, had a high BMI, and didn’t exercise [13]. GrimAge predicts years-to-death (lifespan) using CpG features selected based on their associations with physiological risk factors, stress-related markers, and self-reported smoking pack-years [14]. Age and gender were also included, as they show a modest association with mortality. DunedinPACE leverages the 1972–1973 birth cohort, modeling the Pace of Aging, leveraging elastic net regression and DNA methylation data from blood samples [15]. Epigenetic Timer Of Cancer (epiTOC) focuses on Polycomb group target (PCGT) promoter CpGs, which are typically unmethylated in many stem cells [16], whose model links methylation levels to the rate of stem cell divisions in normal tissue, providing a measure of cancer risks.

More advanced clocks have emerged recently, including Han’s 2020 clock [17], Ying CausalAge [18], and AltumAge [19]. Han’s 2020 aging clock used pyrosequencing to optimally select CpGs [17]. Ying’s CausalAge uses CpGs with the strongest causal relationships with age that previous clocks may not have utilized, using Mendelian randomization to pinpoint CpGs whose methylation directly influences age [18]. Unlike other clocks that use linear regression, AltumAge employs a deep neural network (DNN) model, requiring a larger input of CpG sites (20 K) to prevent underfitting. AltumAge’s unique architecture also captures nonlinear relationships and interactions between numerous CpG sites. AltumAge clock predicted higher age acceleration for many types of cancers [19].

Since each epigenetic clock model was trained on various datasets from different organs and distinct DNAm assay technologies, high variance among the age predictions of previous state-of-the-art clocks exists [20, 21]. To mitigate variance and discrepancies among previously developed clocks and create a reliable and robust epigenetic aging clock, we employed model stacking, a machine learning ensemble technique that combines the predictions of multiple base models, in our case, previously developed DNAm-based epigenetic age clocks, through a higher-level model trained to optimize the final output. By leveraging the strengths of previously developed and well-performing clock models, our EnsembleAge models help reduce both bias and variance. When evaluated on independent datasets, the EnsembleAge clock demonstrated improved overall accuracy across a broad range of ages and tissue types.

Our goal is to build a comprehensive epigenetic aging clock that integrates the strengths of the previously developed clock models by addressing their limitations in accuracy and variance. We developed two EnsembleAge clocks, EnsembleNaive and EnsembleLR, trained and evaluated on the Genotype-Tissue Expression (GTEx) dataset [22], which contains 987 samples of DNA methylation data for blood, breast, kidney, lung, muscle, ovary, prostate, testis, and colon, along with age group annotations.

To contribute meaningfully to both individuals and the aging research community, we have developed a publicly accessible web application that enables users to predict and monitor their biological age. Our EnsembleAge clock service (https://ensemble.epiclock.app/) provides accurate and reliable aging prediction with a user-friendly web interface. By uploading a methylation data file, our service runs ten clock models (eight of previously developed, EnsembleNaive and EnsembleLR) and presents predicted ages via interactive visualization to help users understand the results. Our user interface is specifically designed with senior users in mind, adopting an age-friendly, user-centric approach that is essential for promoting widespread use.

## Methods

We adopted the concept of age acceleration [7, 23] and defined epigenetic age acceleration as the difference between an individual’s epigenetic age and chronological age, which can be formulated as follows;

$$Epigenetic\;Age\;Acceleration\;(years)=Epigenetic\;Age-Chronological\;Age$$

### Model architecture

Model stacking is an ensemble learning technique in which the predictions from multiple base models are combined by a secondary model, often referred to as a meta-learner, to improve overall prediction performance. Rather than relying on a single model, stacking leverages the complementary strengths of several models, each trained independently, by training the meta-learner to find an optimal way to combine their outputs. In our case, each base model corresponds to a pre-trained DNA methylation aging clock, and the meta-learner is our EnsembleAge clock that is trained to produce a final, robust estimate of biological age.

**Fig. 1:**
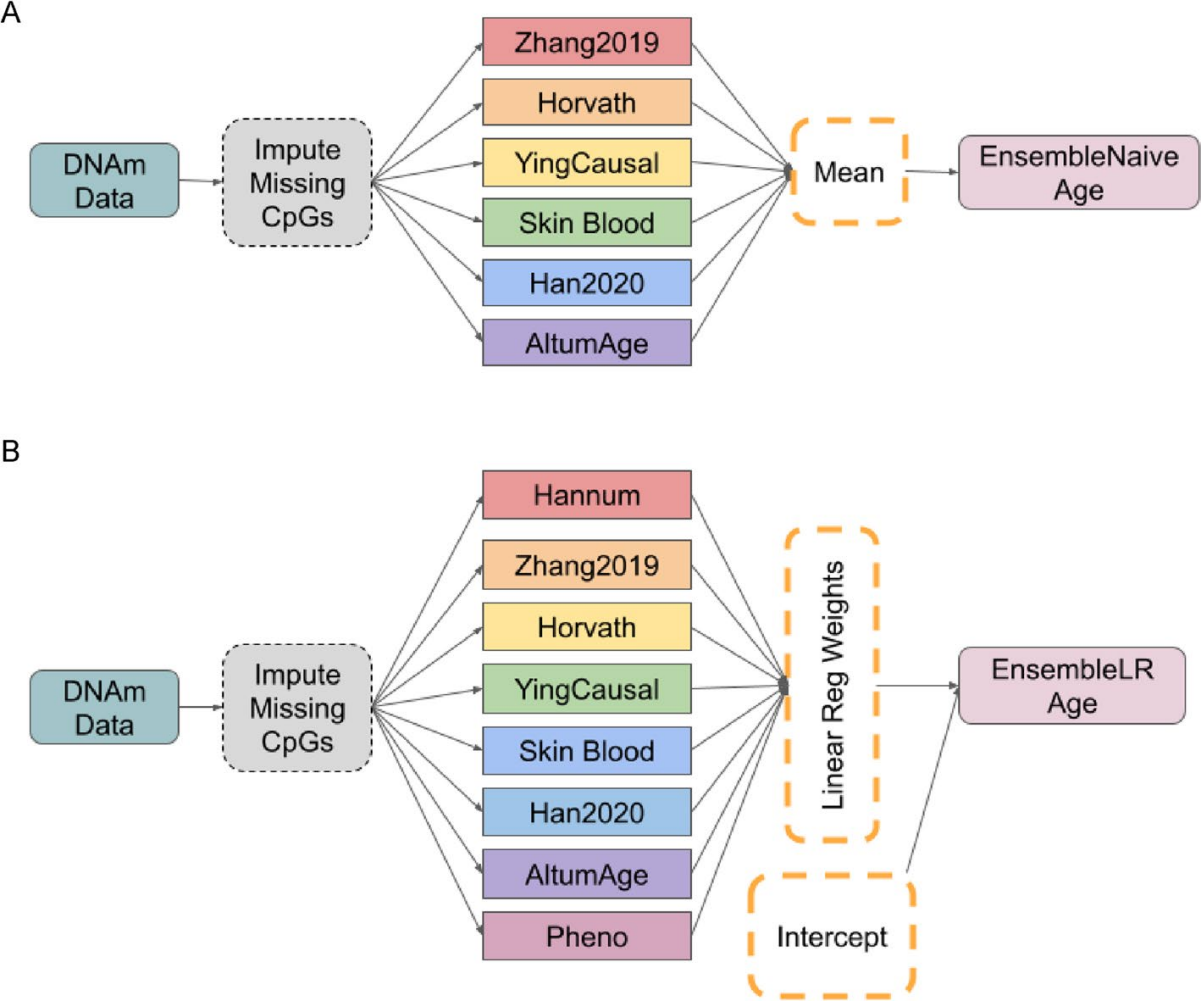
A schematic diagram of EnsembleAge clock models. *EnsembleNaive* uses six DNA methylation (DNAm) aging clocks, while *EnsembleLR* incorporates eight clock models. **A** EnsembleNaive Clock model excluded the Hannum clock from consideration. The Hannum clock shows a high negative weight, which reduces the performance of EnsembleNaive due to its simple averaging approach. **B** EnsembleLR, in contrast, learns optimal weights for each clock. This allows it to optimize the contribution of all eight clocks, balancing their individual performances to produce a more accurate overall prediction

We implemented two types of stacking: uniform stacking (Fig. 1A) and dynamic stacking (Fig. 1B). Uniform stacking assigns equal weights to the predictions from each base model, treating their contributions as equally important. In contrast, dynamic stacking learns model-specific weights, allowing the meta-learner to emphasize more accurate clocks and down-weight less informative ones. This flexibility in dynamic stacking helps reduce variance and bias by smoothing out individual errors and leveraging the strengths of each base model more effectively.

Initially, we used eight pre-trained aging clocks as estimators: Hannum [9], Horvath [7], PhenoAge [13], Skin Blood [10], Ying CausAge [18], Han 2020 [17], AltumAge [19], and Zhang 2019 [11]. Each of these eight estimators uses varying models and training data (Table 1). We aim to integrate the specialized strengths of each of these epigenetic age clocks while compensating for their individual discrepancies.

**Table 1:** Summary of DNAm epigenetic age clocks used for EnsembleNaive and EnsembleLR clock models. Most clocks use linear regression while AltumAge uses deep learning. We utilized clocks developed over the past 12 years, incorporating both unique and shared CpGs, to capture a comprehensive range of biological features and capabilities

Aging Clock	#CpGs	Algorithm	Training Data	Purpose
Hannum	71	linear regression	HM450K Blood	Biological Aging
Horvath	353	linear regression + anti_transform_age	HM450K/27 K Pan-Tissue	Pan-Tissue Aging
PhenoAge	513	linear regression	HM450K Blood Data	Biological Aging, Healthspan
Horvath clock for skin and blood cells	391	linear regression	HM450K & EPIC 850 K Skin, Blood, and Saliva	Biological Aging, Lifespan, Forensics
Zhang	514	linear regression	HM450K & EPIC 850 K Blood & Saliva	Biological Aging
Han	65	linear regression based on pyrosequencing	HM450K Blood	Precise Epigenetic Aging
AltumAge	20318	deep neural network	HM450K/27 K Pan-Tissue	Pan-Tissue Aging
Ying CausAge	586	linear regression	HM450K/27 K Blood	Causality-Enriched Aging

We developed two ensemble aging clocks, EnsembleNaive and EnsembleLR, to predict biological age via DNA methylation data (Fig. 1A-B). EnsembleNaive, as our baseline model, works by using six out of eight of the aging clocks (Altum, Horvath, Skin Blood, Ying CausAge, Han 2020, and Zhang 2019), averaging out all predictions. We excluded PhenoAge clcok from the EnsembleNaive modelbecause it does not directly predict biological age but rather phenotypic age from clinical biomarkers. However, we included DNAm PhenoAge in the EnsembleLR model after observing that its 513 CpGs overlap with those used in other clocks, such as SkinBlood and Horvath’s clock. EnsembleLR, a more advanced model, utilizes all eight epigenetic clock models. We trained a linear regression model with the age estimates from all eight aging clocks.

### Addressing age group imbalance in DNA methylation datasets

We used the GTEx data for the EnsembleLR model training. It contained data generated using the Illumina Infinium MethylationEPIC 850 K BeadChip array for 987 samples across nine different organs, primarily collected from deceased donors. Donors were selected to minimize the presence of disease. Specifically, individuals with cancer or HIV were excluded to focus on normal, healthy tissues, although age distribution is uneven; samples aged 40–60 years were much more common than younger and older samples were [22, 24]. To account for age group imbalance and ensure that our model accurately predicts epigenetic age on data from a wider age range of 20–80, we used the oversampling technique, known as synthetic minority oversampling technique (SMOTE) [25, 26].

SMOTE generates synthetic training samples for fewer occurring age groups in our dataset to ensure unbiased training. SMOTE generates synthetic samples for underrepresented age groups by selecting a sample from a minority group and identifying its nearest neighbors in the training data. Then, it creates a new sample by taking a weighted average of that sample and one of its neighbors. This way, the synthetic data point lies along the line connecting the two real points in the methylation feature space. Unlike simple duplication, this method produces more varied and realistic examples. In our case, this approach helps fill gaps where few individuals are present—such as in the 20–40 and 60–80-year-old (yo) age ranges—allowing the model to learn age patterns more evenly across the entire 20–80 yo span, rather than being biased toward the densely populated 40–60 yo range.

### Training procedure for the EnsembleLR prediction model

We split the oversampled GTEx dataset into training and test data with an 80/20 split ratio. We then applied an L2 linear regression model with alpha = 0.1 to the training data to learn the optimal weights for each epigenetic clock, as well as an intercept, to compute the final EnsembleLR predicted age. The EnsembleLR coefficients after being trained with L2 regularization are as follows: AltumAge (0.34), Han 2020 (−0.21), Hannum (−0.52), Horvath (0.28), Skin Blood (0.19), Pheno (−0.02), YingCausal (−0.09), and Zhang 2019 (0.56), with an intercept of 19.28. Notably, the coefficient assigned to PhenoAge was −0.02, the smallest absolute value among all clocks, indicating minimal influence on the final prediction. Nonetheless, its inclusion may help reduce overfitting by providing an additional noise-like component.

### Relative contributions of individual clocks in EnsembleLR

The coefficients learned by EnsembleLR reflect how much each aging clock contributes to robust epigenetic age prediction across the diverse GTEx tissue samples. Clocks such as Zhang 2019 (0.56) and AltumAge (0.34) received the highest positive weights, indicating that their predictions closely track chronological age in a generalizable way. Zhang 2019 was trained on both blood and saliva samples using data from the 850 K array, likely making it well-suited for GTEx's multi-tissue setting. AltumAge, a deep learning model with over 20,000 CpGs, may provide a nonlinear, high-capacity representation of aging signals that complements traditional linear clocks.

In contrast, Hannum (−0.52) and Han 2020 (−0.21) received negative coefficients, implying that their predictions tended to introduce systematic biases when applied to GTEx data. These models were trained exclusively on blood-derived samples and with fewer CpGs, which limits their generalizability across tissues like skin, brain, or muscle. The regression model effectively learns to “correct” these biased contributions by downweighting them to reduce overall error.

Overall, these weighting patterns demonstrate that EnsembleLR systematically prioritizes clocks trained on pan-tissue data, with broad CpG coverage and generalizable modeling approaches, while assigning lower weights to clock models constrained by narrow biological scope or specific design purposes. This selective weighting enhances our EnsembleAge clock model’s ability to provide accurate and biologically robust epigenetic age estimates across diverse samples.

### Imputations of missing data

As a preprocessing step, our EnsembleAge clock models use ‘IterativeImputer’ from scikit-learn to fill in any missing CpG methylation values required for age prediction [27]. This multivariate imputer works by modeling each CpG site with missing values as a regression problem, leveraging the observed methylation data of other CpGs. In each iteration, a regression estimator, BayesianRidge, predicts the missing values of one CpG site using all other measured sites as inputs. This process continues for every CpG site with missing data, iteratively refining the imputations over several rounds until convergence. In our implementation, we specifically set the imputer to run with `max_iter = 10` and `random_state = 0` to control the number of refinement cycles and ensure reproducibility. The imputer starts with an initial estimate, the feature mean, and repeatedly updates the missing values by learning a linear relationship between observed and missing CpG methylation values.

Imputation is applied at the time of prediction using the user's data for our EnsembleAge clock web service, in case any feature CpGs are missing. After imputation of the missing input data, our EnsembleAge clock service provides age estimates predicted by each of the clock models selected by a user.

### EnsembleAge clock web service

**Fig. 2:**
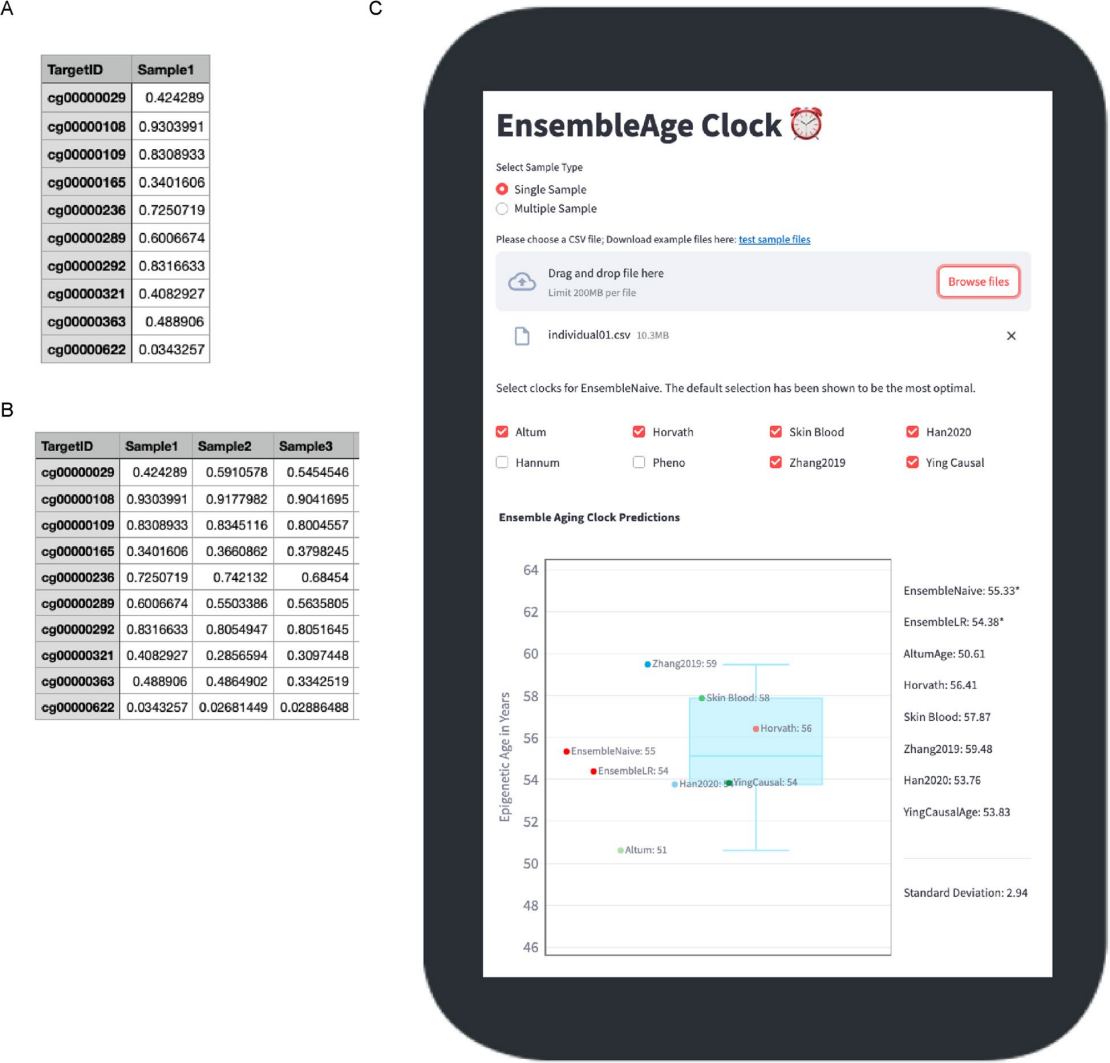
Input formats and user interface of the EnsembleAge clock web service. **A** DNA methylation input data format for a single sample. The data is provided in comma-separated values (CSV) format, with each row representing an individual CpG site. **B** DNA methylation input data format for multiple samples. The data is also in CSV format, where each row corresponds to a CpG site and each column represents an individual sample. **C** The EnsembleAge clock web service is universally accessible and provides a user-friendly platform for epigenetic age prediction across all age groups. Upon uploading DNA methylation data, the service automatically imputes missing values, applies two clock models, and presents the predicted ages in a clear and interactive boxplot

With our EnsembleAge models, we implemented a web application service (https://ensemble.epiclock.app) for single and multiple samples. Users can upload their own methylation data in a CSV format, consisting of cgID and methylation ratio, which is represented as a beta value, calculated as M/(M + U), where M denotes methylated molecules and U denotes unmethylated molecules. The methylation values are already normalized and therefore range between 0 and 1, so no further normalization is required. An input file for a single sample of methylation data should consist of two columns: the CpG ID (cgID) in the first column and the methylation ratio in the second column (Fig. 2A). For multi-sample uploads, the format should follow that of a single sample, with additional columns added after the second column to accommodate subsequent samples (Fig. 2B). The app contains checkboxes for eight different clocks, which is an option for EnsembleNaive (Fig. 2C). Users can select which clocks to include in the EnsembleNaive prediction. While we recommend using all six clocks, the choice is ultimately up to the user.

## Results

### Low errors across clock models and organ aging

**Fig. 3:**
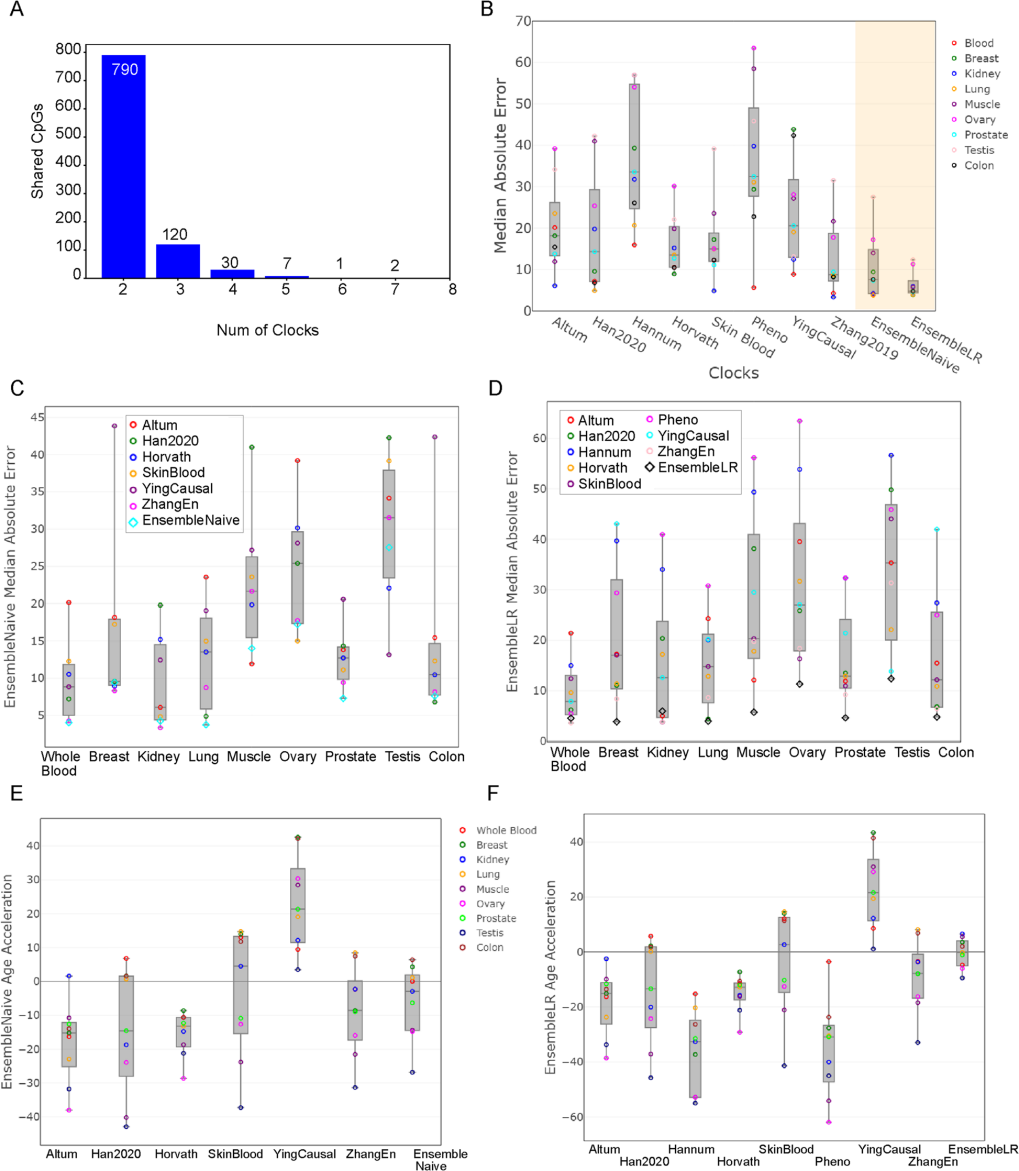
Performance and CpG overlap of epigenetic clocks across multiple tissues. **A** Histogram showing CpG overlap among the eight epigenetic clocks used. No CpG sites are shared across all eight clocks, highlighting the unique contributions of each. However, overlapping CpGs emphasize their importance, as recognized by the EnsembleAge clock models. **B** Performance comparison of ten epigenetic clocks using DNA methylation data from nine organs in the GTEx dataset. Our EnsembleAge models (EnsembleNaive and EnsembleLR) achieved the lowest median absolute errors (MeAE) and the smallest variance across all organs, demonstrating superior reliability. **C** EnsembleNaive model achieved the lowest MeAE in whole blood, lung, and prostate, and performed consistently well across other organs. **D** EnsembleLR model demonstrated the lowest MeAE in breast, lung, muscle, ovary, prostate, testis, and colon, and the second-lowest in whole blood. **E** In healthy normal samples, age acceleration (AA) is expected to be close to zero for healthy organs and tissues if the clock model performs well. The EnsembleNaive model produces AA values near zero, indicating its strong performance across the nine organs. **F** The EnsembleLR model also demonstrates superior age prediction accuracy for test data, with AA values close to zero and minimal variation across healthy organs

To improve accuracy and reduce variance, we leveraged eight previously developed DNAm clocks, including the Hannum, Horvath, Horvath Skin Blood, YingCausal, Zhang2019, Pheno, Altum, and Han2020 clock models. We analyzed CpGs from the eight clock models to harness the strengths of each DNAm aging clock, smoothing out individual variances and providing a more robust estimation of biological age (Fig. 3A, Supplementary Tables 1–6). A total of 790 CpGs are found in exactly two clocks, 120 CpGs are found in exactly three clocks, 30 CpGs are found in exactly four clocks, 7 CpGs are found in exactly five clocks, one CpG is found in exactly six clocks, two CpGs are found in seven clocks, and no CpGs are found in all eight clocks. The two CpGs found in at least one 7-clock combination were cg19722847 and cg09809672. cg19722847 is located in the ELOVL2 gene, a well-established epigenetic biomarker of aging that shows consistent hypermethylation with increasing age across multiple tissues [28]. cg09809672 lies within the FHL2 gene, which is implicated in cell senescence and age-related pathologies, including cardiovascular and neurodegenerative diseases [29]. The presence of these two highly age-associated CpGs—shared by seven out of eight clocks—underscores their strong predictive power and importance in estimating biological age, highlighting their key role in accurately modeling epigenetic aging [30, 31]. Since these previous clocks have all demonstrated strong performance, incorporating unique CpGs with distinct weights from each clock is beneficial for our EnsembleAge models.

We evaluated the magnitude of error in our Ensemble clocks via the median absolute error (MeAE) and mean absolute error (MAE) (Supplementary Tables 7–8). The GTEx project focuses on non-diseased human tissues, so our goal was to reduce our clocks’ MeAE and MAE, as biological age should closely align with chronological age in healthy tissues [22, 32] (Supplementary Tables 7-8). For whole blood, lung, and prostate samples in GTEx, EnsembleNaive had the lowest MeAE out of the six clocks that it uses for its prediction, including the Altum, Han2020, Horvath, Horvath Skin Blood, YingCausal, and Zhang2019 clock models (Fig. 3B). EnsembleNaive also maintains the MeAE of less than five years for whole blood, lung, and kidney data. In addition, for the breast, prostate, and colon data, EnsembleNaive still performs best, maintaining an MeAE of under ten years (Fig. 3B). The only organs where EnsembleNaive performed poorly were muscle, ovary, and testis, whose training data were primarily whole blood (Fig. 3C).

One of the motivations for building EnsembleLR was to develop a model for data generated via the Illumina Infinium MethylationEPIC 850 K Beadchip array. As a result, our MeAE for EnsembleLR is consistently much lower than EnsembleNaive’s MeAE across all organs. The data that we tested on EnsembleLR were not used for training and was not synthetically generated by SMOTE. This new and improved model has the lowest MeAE out of the eight clocks: Altum, Han2020, Horvath, Skin Blood, YingCausal, Zhang2019, Pheno, Hannum, for all organs except whole blood, kidney, and lung (Fig. 3D). Our MeAE is under 6 years for all organs except for the ovary and testis, which both remain under 13 years (Supplementary Tables 7–8). Most notably, this improved EnsembleLR reduces the testis MeAE to 13 years from 27 years with EnsembleNaive and reduces the MeAE by nine and six years for the muscle and ovary, respectively, in comparison to EnsembleNaive.

When epigenetic age clock models are built, only healthy, normal tissues are used for training and testing. Therefore, it is ideal for the median absolute error (MeAE) to be close to zero across samples and datasets. As shown, our two models—EnsembleNaive and EnsembleLR—demonstrate the most stable Epigenetic Age Acceleration (EAA) values, consistently centered around zero (Fig. 3E-F, Supplementary Table 9). All samples used in this analysis were derived from healthy tissues across nine different organs.

### Improving prediction robustness

To compare the predicted age (y-axis) of our aging clocks with the true chronological age (x-axis) of the samples, we plotted these values and calculated the line of best fit. The slope of this line indicates the robustness of our EnsembleAge clock predictions, with a slope of 1 being ideal. ​​Each clock shows a unique pattern in age predictions that the summarized MeAE or MAE values could not previously explain.

**Fig. 4:**
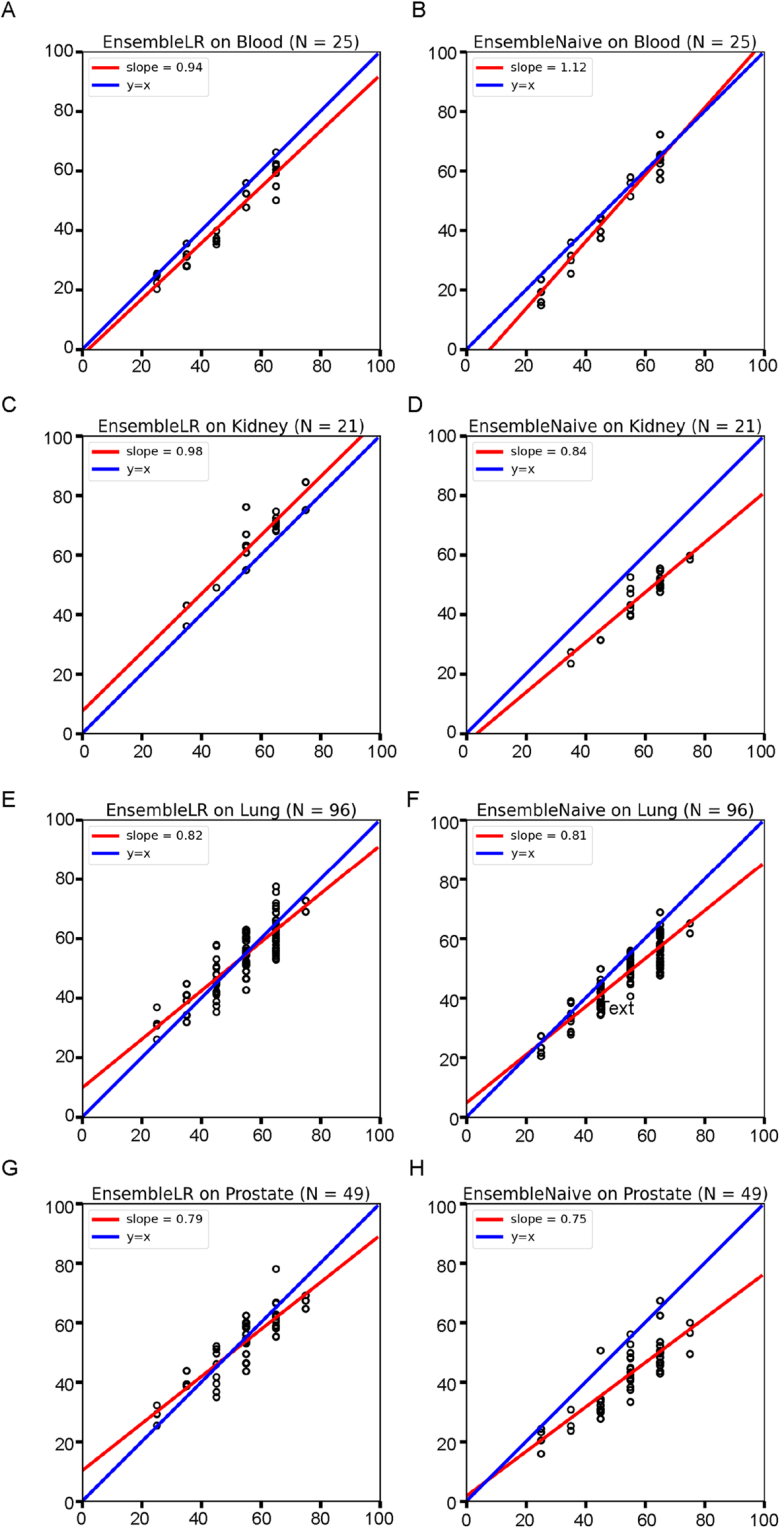
Validation of EnsembleAge clocks using GTEx data. We performed in silico validation using the GTEx dataset for four organs — (**A**-**B**) whole blood, (**C**-**D**) kidney, (**E**–**F**) lung, and (**G**-**H**) prostate — to demonstrate the robustness of our EnsembleLR and EnsembleNaive clocks. In the plots, the x-axis represents chronological age, while the y-axis shows the age predicted by the EnsembleAge Clocks. Each point represents a sample, with the red line indicating the line of best fit and the blue line representing the ideal y = x reference. Both EnsembleNaive and EnsembleLR models performed best in whole blood (slopes of 0.94 and 1.12, respectively), followed by kidney (slopes of 0.98 and 0.84). Age predictions for the prostate deviated more from the diagonal line compared to other organs, likely due to a relative lack of training data. Overall, EnsembleLR outperformed EnsembleNaive across the tested organs

Compared with the eight individual clocks it uses and EnsembleNaive, EnsembleLR, which is optimized on GTEx data, yields many improved slopes (Fig. 4A-H). The slopes for whole blood are 0.94 and 1.12, the closest to 1 (Fig. 4A-B). EnsembleLR and EnsembleNaive perform exceptionally well for the kidney, with slopes of 0.98 and 0.84, respectively (Fig. 4C-D). These improved slopes indicate that linear regression is better at reducing the prediction variance than average predictions are. EnsembleLR also performed well for the lung, with slopes of 0.82 and 0.81 (Fig. 4E-F), and for the prostate, with slopes of 0.79 and 0.75 (Fig. 4G-H). These slopes reveal that each of the eight individual clocks has different prediction trends for certain organs, whether underprediction or overprediction. Nonetheless, both EnsembleAge clocks demonstrate the ability to balance these variances, maximizing accuracy across all organs from GTEx methylation dataset.

### Correlation between whole blood CpG methylation and chronological age

**Fig. 5:**
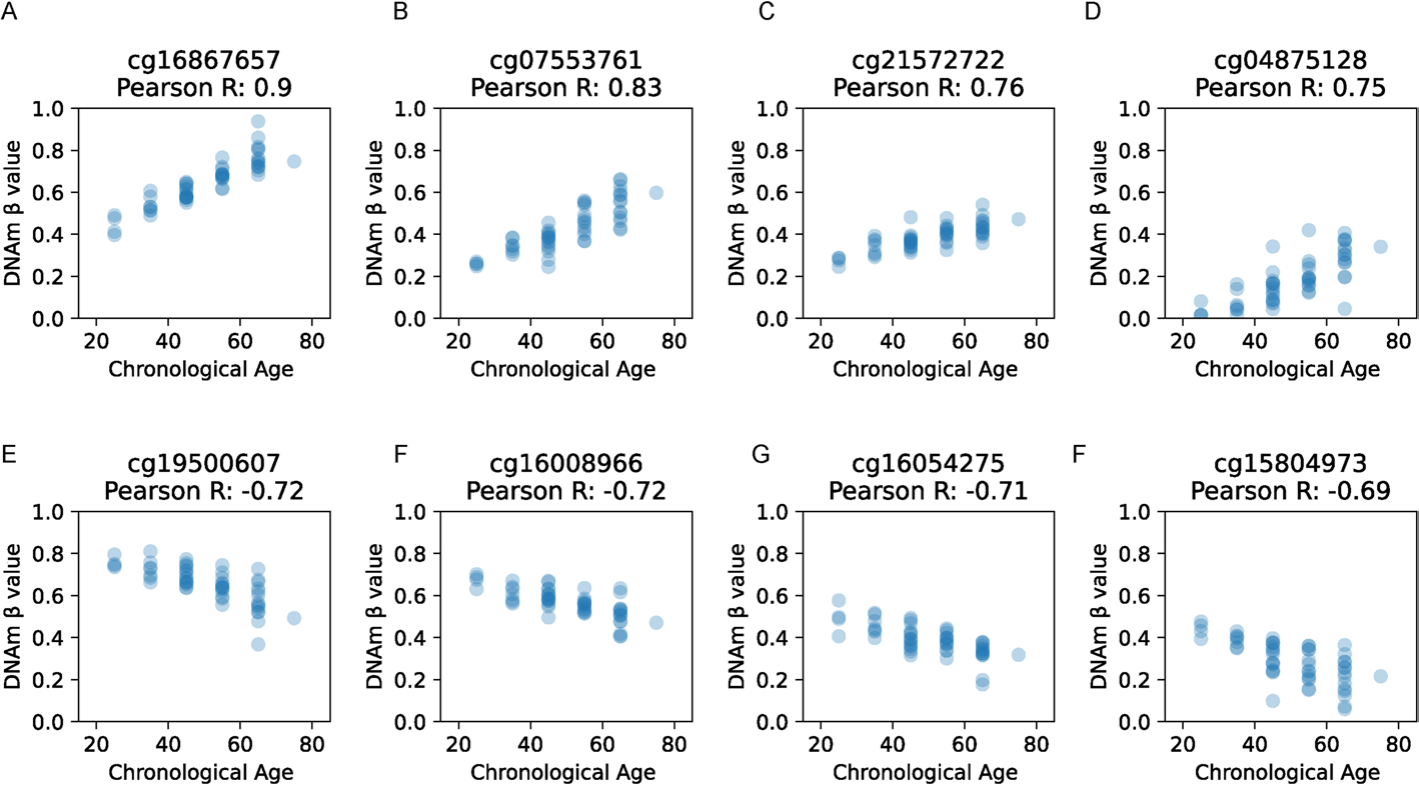
Association between chronological age and whole blood DNA methylation at specific CpG sites. Each plot illustrates the relationship between chronological age and DNA methylation levels for a specific CpG site. Panels (**A**–**D**) show the four CpG sites most positively correlated with age, while panels (**E–H**) display the four most negatively correlated. Overall, positively correlated CpGs exhibit a stronger association with age compared to negatively correlated ones

To validate the selection of CpGs from each clock, we analyzed the CpGs from all eight clocks to measure the correlation between DNAm and age. We calculated Pearson correlation coefficients between the DNA methylation ratios across samples and their chronological ages (Fig. 5A-F, Supplementary Table 10). We then identified the top four positively correlated CpGs with age (Fig. 5A-D) and the top four negatively correlated CpGs (Fig. 5E-H) for blood GTEx samples to confirm their individual contributions and relevance. This step also helped illuminate the direction and degree of strength of various CpGs’ methylation ratio and age association, and furthermore allowed us to investigate top correlated CpGs’ involvements in specific biological processes, reinforcing their interpretability in biological age prediction.

With their presence across many of EnsembleAge’s base model clocks, it emphasizes their stacked contributions to the EnsembleAge clocks to further improve accuracy. The strong correlation between these CpGs’ blood DNAm data and chronological age in the GTEx dataset also demonstrates the high fidelity of our clock and previous clocks' analysis of blood DNAm data. To better highlight their individual contributions, the specific biological relevance and age-associated methylation pattern of each CpG is as follows.

Cg16867657 (PCC: 0.90) is located in the Elongation of Very Long Chain Fatty Acids Protein 2 (ELOVL2) gene and is one of the most consistently replicated age-associated CpG sites, showing a near-perfect positive correlation with age across multiple tissues [33]. Cg07553761 (PCC: 0.83), which is linked to the TRIM59 gene, shows strong age-associated methylation changes and has been identified among key CpGs that are predictive of aging in multiple regression-based epigenetic clocks [34]. Cg21572722 (PCC: 0.76) is also located in ELOVL2 and appears to be one of the top differentially methylated sites associated with age in whole blood [35]. The ELOLV2 gene plays a key role in elongating fatty acids involved in lipid metabolism. Cg04875128 (PCC: 0.75) is located within the FHL2 gene and exhibits strong age-associated methylation across tissues, making it a widely used feature in aging clocks because of its robust age correlation [36].

Cg19500607 (PCC: −0.72) is located in the HTR4 gene which encodes the 5-hydroxytryptamine (serotonin) receptor 4, which has been shown to play roles in neurodevelopment and neural function, and its signaling is involved in pathways relevant to aging processes such as neurogenesis, synaptic plasticity, and cognitive performance [37]. Cg16008966 (PCC: −0.72), found in the SLC12A5 gene, shows methylation patterns significantly associated with aging and cognitive health outcomes in population epigenetics studies [38]. Cg16054275 (PCC: −0.71), associated with the GPR15 gene, exhibits decreased methylation with age and has also been identified in environmental epigenetic studies, further supporting its relevance in aging and immune system regulation [39]. Cg15804973 (PCC: −0.69), located in the promoter region of MAP3K5, is negatively correlated with age and has been linked to aging-related cell stress and apoptosis pathways [40, 41]. Together, these eight CpGs, spanning genes involved in lipid metabolism, immune regulation, neural function, and stress response, form a biologically diverse and age-sensitive panel. Age-related diversity, including positive and negative correlation, underscores their collective strength in enhancing the precision and robustness of our EnsembleAge clock models in capturing biologically meaningful age differences in tissues affected by various factors [42]. Given the strong correlation of these CpGs to methylation changes associated with aging, alterations at these sites may serve as indicators of epigenetic age acceleration or as potential risk factors for age-related diseases.

### Clinical and forensic applications of reliable and robust EnsembleAge clock models

**Fig. 6:**
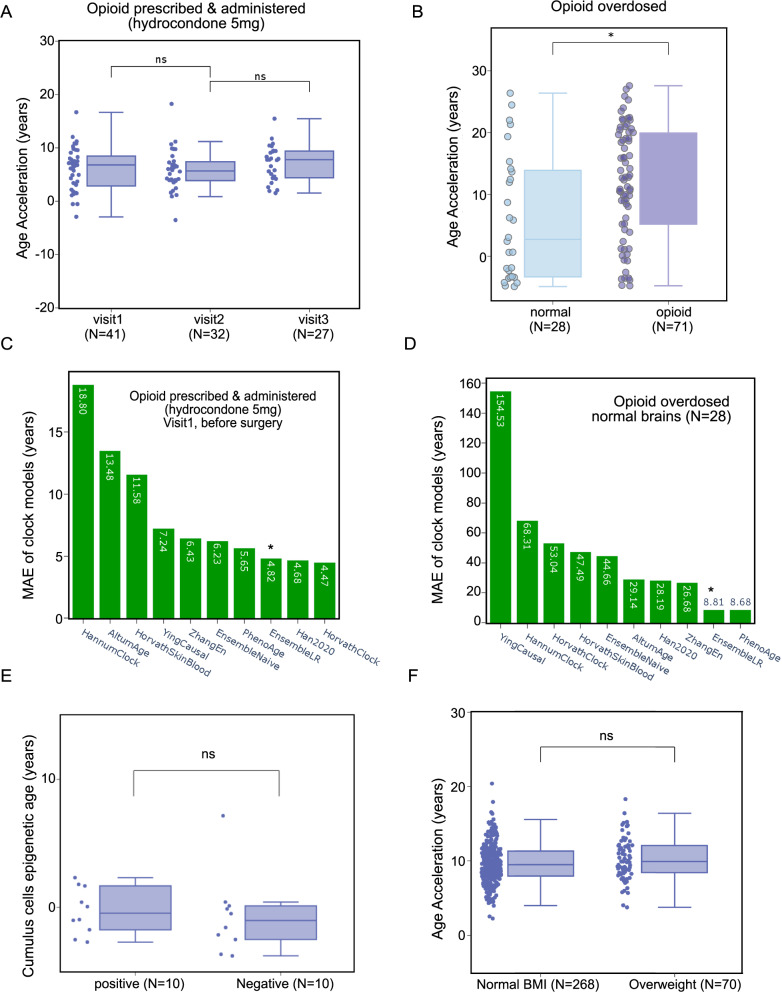
Applications of the EnsembleAge clock models across multiple clinical and forensic datasets (GSE151485, GSE164822, GSE144664, GSE73103). We applied our EnsembleAge clock models (EnsembleNaive and EnsembleLR) to four publicly available DNA methylation datasets. **A** GSE151485*:* We applied our EnsembleAge clock models to data from 33 opioid-naïve participants who underwent standard dental surgery followed by short-term opioid self-administration. Saliva samples were collected at three time points: before surgery (visit 1), and at two post-surgery visits - 2.7 ± 1.5 days (visit 2) and 39 ± 10 days (visit 3) after discontinuation of opioid analgesics. No significant age acceleration was observed at any time point. **B** GSE164822: We applied our EnsembleAge clocks to dorsolateral prefrontal cortex tissue samples obtained from deceased individuals, including 71 who died from acute opioid intoxication and 28 group-matched controls. The opioid intoxication group exhibited approximately 10 years of age acceleration compared to the control group. **C**–**D** Mean Absolute Error (MAE) analysis demonstrates that the EnsembleLR model consistently achieves low prediction error, supporting its robustness and the superiority of model stacking ensemble method. **E** GSE144664: Cumulus cells were collected from women undergoing intra-cytoplasmic sperm injection (ICSI) cycles to compare epigenetic ages between samples resulting in positive versus negative pregnancy outcomes. Although cumulus cells from pregnancy-positive cases showed signs of greater maturation compared to those from unsuccessful cases, the small sample size and high sensitivity of methylation ratio changes prevented the results from reaching statistical significance. **F** GSE73103: We applied our EnsembleAge clock models to obesity-related HM450 methylation data and found no evidence of age acceleration in the overweight cohort (*N* = 70) compared to the normal BMI cohort (*N* = 269)

We applied EnsembleNaive and EnsembleLR to DNA methylation data from short-term and well-administered dosages such as therapeutic opioids (GSE151485) [43]. We estimated epigenetic methylation data from saliva samples collected before surgery and at the two follow-up visits after surgery. The therapeutic opioid, hydrocodone 5 mg, was self-administered after surgery and before the first visit. Epigenetic age acceleration was not significantly different before and after patients took the prescribed opioid at either the second visit, 1–6 days later, or the third visit, 28–74 days later (Fig. 6A, Supplementary Table 11). Opioid abuse presents a significant risk to individuals in the United States, and epigenetic changes are emerging as key potential biomarkers of opioid addiction. We applied EnsembleNaive and EnsembleLR models to DNA methylation data from tissue samples collected from the dorsolateral prefrontal cortex of deceased individuals, including 71 opioid overdosed users and 28 normal controls (GSE164822) [44]. Our analysis revealed a statistically significant increase in epigenetic age of more than 10 years in the dorsolateral prefrontal cortex samples from individuals who abused opioids (Fig. 6B, Supplementary Table 12). GSE164822 samples were collected from the brain samples of the deceased, where the age distribution is between 10—50 years old. Although epigenetic age is underestimated in the older aged population, we were able to observe significant age acceleration in the opioid overdose cohort. MAE analysis demonstrates our EnsembleLR model reliably predicted epigenetic age and capture epigenetic age acceleration across the two datasets (Fig. 6C-D).

We applied our EnsembleAge clock models to cumulus cells, highly specialized ovarian somatic cells that support the growth, development, and maturation of the oocyte. Cumulus cells were collected from women undergoing intra-cytoplasmic sperm injection (ICSI) cycles to compare epigenetic ages between samples resulting in positive versus negative pregnancy outcomes (GSE144664) [45]. Although cumulus cells from pregnancy-positive cases exhibited signs of greater maturation compared to those from unsuccessful cases, the small sample size and high sensitivity of methylation ratio changes prevented the results from reaching statistical significance (Fig. 6E, Supplementary Table 13). We applied our EnsembleAge clock models to obesity-related methylation data (GSE73103) [46], which includes an overweight cohort (*N* = 70) and a normal BMI cohort (*N* = 269). Little evidence of age acceleration was observed in the overweight group compared to the normal BMI group (Fig. 6F, Supplementary Table 14). Four applications highlight the significant potential of EnsembleAge clock models for both clinical and forensic usage.

## Discussion

Our EnsembleAge clocks demonstrate moderate and reduced variance, which contributes to more reliable epigenetic age prediction. We propose a pan-tissue clock that builds upon previously developed clocks and integrates data from multiple DNA methylation platforms, including the HumanMethylation450 (HM450K) BeadChip and the Infinium MethylationEPIC arrays. Although the EPIC array has largely replaced the HM450K array in recent epigenetic research, much of the training data of earlier clocks was based on the HM450K platform. It is likely that previously developed clock models were biased toward the HM450K platform. Notably, discrepancies between the two technologies have been reported [47, 48]. Our EnsembleAge clock models leverage eight existing clocks, all originally trained on HM450K data, with two also incorporating EPIC array data. We trained the EnsembleLR model using GTEx DNA methylation data generated on the EPIC platform, potentially enhancing compatibility with other EPIC-based datasets.

A major limitation in creating a pan-tissue aging clock lies in the lack of training data for machine learning models, which require a greater number and variety of samples to be precise and fine-tuned. Both EnsembleLR and EnsembleNaive clock models perform exceptionally well on blood data, which is the tissue on which their eight subsidiary clocks were trained. Our model stacking approach effectively reduces the variance among the age predictions of subsidiary models.

DNAm-based age prediction has valuable applications in forensic science, particularly for gaining information about unknown individuals who have left DNA at crime scenes. This supports forensic DNA phenotyping, as age is a critical trait when narrowing down potential suspects. Estimating age from crime scene DNA can be highly informative, especially when combined with other genetic information such as ancestry. For forensic applications, highly reliable methods that require a minimal number of markers are essential, as the DNA available is often degraded or damaged. For example, Weidner et al. developed a model based on three CpG sites [49], while the VISAGE Consortium developed models using six to eight CpG sites [50]. However, none of these models are publicly available, as their coefficients (weights) remain private. As a result, our team was unable to include them in our analysis or EnsembleAge clock models. Nevertheless, this remains an exciting area for future development, particularly for building dynamic age clocks tailored to forensic needs. While our current model has not yet been applied to actual forensic casework, it offers a robust methodological foundation. Future work will focus on testing the model under forensic-like conditions, such as degraded samples, mixed body fluids, and low-DNA-input contexts. We plan to incorporate mock forensic exhibits in subsequent validations and explore reduced-feature versions of the clock more suitable for field deployment. Publicly available forensic methylation datasets and collaborations with forensic laboratories will be essential to ensure practical applicability.

Importantly, while EnsembleLR is built upon linear regression, future iterations could explore non-linear meta-learners (e.g., gradient boosting, random forests, or neural networks) to capture potential interactions between CpGs in complex epigenetic aging process, particularly in non-linear aging acceleration among organs. Prior work like AltumAge already demonstrates the power of deep learning to model such nonlinearity while employing the largest number of CpG markers (> 20 K). Building interpretable nonlinear ensemble models, optimizing the number of CpGs, could be a valuable direction for enhancing biological age prediction.

Our web service allows people to check their biological age by uploading their own methylation data files. On our web application service (https://ensemble.epiclock.app/), the EnsembleAge clock’s age predictions can indicate whether users may be at risk for age-related diseases, based on whether their predicted biological age exceeds their chronological age. In conclusion, convenient computational tools, such as the EnsembleAge clock web service, are essential for consistently and accurately tracking an individual’s epigenetic age, thereby monitoring health status and promoting healthy aging, and a healthy lifestyle.

## Supplementary Information

Supplementary Material 1.

## Data Availability

For performance evaluation and validation, we used DNA methylation data from the GTEx dataset (GSE213478). In addition, four external datasets were used for further validation and application. To assess epigenetic age acceleration in patients prescribed opioids, we analyzed GSE151485. DNA methylation data from dorsolateral prefrontal cortex tissues of individuals with opioid use disorder are available in GSE164822. Methylation data related to assisted reproductive technology (ART) were obtained from GSE144664, and obesity-related methylation data from GSE73103.
